# High Fat Diet Subverts Hepatocellular Iron Uptake Determining Dysmetabolic Iron Overload

**DOI:** 10.1371/journal.pone.0116855

**Published:** 2015-02-03

**Authors:** Paola Dongiovanni, Claudia Lanti, Stefano Gatti, Raffaela Rametta, Stefania Recalcati, Marco Maggioni, Anna Ludovica Fracanzani, Patrizia Riso, Gaetano Cairo, Silvia Fargion, Luca Valenti

**Affiliations:** 1 Internal Medicine and Metabolic Diseases, Fondazione IRCCS Ca’ Granda Ospedale Policlinico, Milano, Italy; 2 Department of Food, Environmental and Nutritional Sciences, Università degli Studi di Milano, Milano, Italy; 3 Preclinical Surgical Research Center, and Surgery, Fondazione IRCCS Ca’ Granda Ospedale Policlinico, Milano, Italy; 4 Department of Pathophysiology and Transplantation, Metabolic Liver Diseases Research Center, Università degli Studi di Milano, Milano, Italy; 5 Department of Biomedical Sciences for Health, Università degli Studi di Milano, Milano, Italy; 6 Pathology, Fondazione IRCCS Ca’ Granda Ospedale Policlinico, Milano, Italy; Lady Davis Institute for Medical Research/McGill University, CANADA

## Abstract

Increased serum ferritin associated with mild hepatic iron accumulation, despite preserved upregulation of the iron hormone hepcidin, is frequently observed in patients with dysmetabolic overload syndrome (DIOS). Genetic factors and Western diet represent predisposing conditions, but the mechanisms favoring iron accumulation in DIOS are still unclear. Aims of this study were to assess the effect a high-fat diet (HFD) on hepatic iron metabolism in an experimental model in rats, to further characterize the effect of free fatty acids on iron metabolism in HepG2 hepatocytes *in vitro*, and to assess the translational relevance in patients with fatty liver with and without iron accumulation. Despite decreased uptake of dietary iron, rats fed HFD accumulated more hepatic iron than those fed regular diet, which was associated with steatosis development. Hepatic iron accumulation was paralleled by induction of ferritin, in the presence of preserved upregulation of hepcidin, recapitulating the features of DIOS. HFD was associated with increased expression of the major iron uptake protein Transferrin receptor-1 (TfR-1), consistently with upregulation of the intracellular iron sensor Iron regulated protein-1 (IRP1). Supplementation with fatty acids induced TfR-1 and IRP1 in HepG2 hepatocytes, favoring intracellular iron accumulation following exposure to iron salts. IRP1 silencing completely abrogated TfR-1 induction and the facilitation of intracellular iron accumulation induced by fatty acids. Hepatic TfR-1 mRNA levels were upregulated in patients with fatty liver and DIOS, whereas they were not associated with liver fat nor with inflammation. In conclusion, increased exposure to fatty acids subverts hepatic iron metabolism, favoring the induction of an iron uptake program despite hepatocellular iron accumulation.

## Introduction

Increased serum ferritin associated with mild iron accumulation is frequently observed in patients with metabolic syndrome and insulin resistance, whose hepatic manifestation is nonalcoholic fatty liver (NAFLD) [1–5], and this syndrome has been termed dysmetabolic iron overload (DIOS) [6,7].

Recent studies suggest that increased iron stores play a causal role in the pathogenesis of insulin resistance and dyslipidemia by altering adipose tissue function [6,8–10], whereas iron depletion improves insulin sensitivity [11,12]. In patients with NAFLD, ferritin levels and hepatic iron deposition are associated with the severity of liver damage [13–16], and hyperferritinemia and macrophage iron accumulation promote vascular damage [17,18].

In contrast to hereditary hemochromatosis, the hormone hepcidin, that inhibits intestinal iron absorption and recycling from macrophages, seems up-regulated concomitantly with increased iron stores in DIOS [19]. Conversely, decreased iron mobilization from hepatic macrophages due to downregulation of the iron exporter and hepcidin target Ferroportin-1 (Fp-1) has been observed in patients with NAFLD complicated by inflammation [20–22]. However, whether these molecular alterations are secondary to obesity, steatosis, or qualitative changes in dietary components is presently unknown. Indeed, genetic factors and Western diet represent predisposing conditions [23], but the mechanisms favoring iron accumulation in DIOS are still unclear.

To help elucidating these issues, aims of this study were to assess the effect a high-fat diet (HFD) on hepatic iron metabolism first in an experimental model in rats [24], then to allow a better functional characterization of the findings in HepG2 hepatocytes *in vitro,* and finally to evaluate their translational relevance in patients with NAFLD with and without DIOS.

## Materials and Methods

### Animal model

Six-week male Sprague-Dawley rats (180–200 g) were purchased from Charles River (Charles River, Calco, Italy) and maintained at the Preclinical Research Center of the Fondazione IRCCS Ca’ Granda Ospedale Policlinico Milano, Italy, in compliance with the Principles of Laboratory Animal care (NIH publication no. 86–23, 1985). The experimental protocol was approved by the Italian Ministry of Health Review Board (prot. 13/10). Animals were housed at constant room temperature (23°C) under 12 hours light/dark cycles with *ad libitum* access to water.

In order to assess the effect of fatty acids on iron metabolism, we considered three experimental groups: 1) rats fed regular chow diet as controls, 2) high fat diet (HFD), and 3) HFD plus iron supplementation (to counterbalance the reduced iron content in HFD chow).

Rats were fed for 12 weeks a standard chow (5% of energy derived from fat: saturated/unsaturated fat 0.9/4.6 g/100 g diet with n-6-to-n-3 poly-unsaturated fatty acids ratio of 11.3; 18% from protein and 77% from carbohydrates; 3.3 Kcal/g, iron content 8.5 μg/100 mg diet; control, n = 6), or HFD (58% of energy derived from fat, saturated/unsaturated fat 30.4/5.3 g/100 g diet with n-6-to-n-3 poly-unsaturated fatty acids ratio of 85.9; 18% from protein and 24% from carbohydrates; 5.6 Kcal/g as described [24], except for the lack of fructose supplementation in water, iron content 0.74 μg/100 mg diet, n = 6), or HFD plus s.c. iron administration at week 8 (250 mg iron sulfate, n = 6). Rats were sacrificed under anesthesia and samples of livers (right sections) were immediately harvested and flash frozen in liquid nitrogen. A portion of liver tissue (left section) was placed in a formaldehyde solution and routinely processed for histological assessment. Sections were stained with hematoxylin-eosin (H&E), and Perls’ Prussian blue staining for iron. Blood hematocrit and serum iron levels were measured by standard methods. Hepatic iron concentration (HIC) and hepatic dietary content were measured by atomic absorption spectrometry [25]. Serum insulin was measured by a commercial ELISA kit (Millipore, Billerica, MA). Serum hepcidin was evaluated by a commercial EIA kit (Bachem, San Carlos, CA).

### Isolation of Tissue and Cellular RNA and quantitative Real Time PCR

RNA was isolated both from tissues and cells by a guanidinium isothiocyanate-phenol-chloroform procedure using Trizol reagent (Life Technologies, Carlsbad, CA). First-strand cDNA was synthesized starting from 1 μg of total RNA using the VILO random examers synthesis system (Life Technologies). Quantitative real time PCR (qRT-PCR) was performed by using the SYBR green chemistry (Fast SYBR Green Master Mix, Life Technologies, Carlsbad, CA). All the reactions were delivered in triplicate by the 7500 Fast Real-Time PCR system (Life Technologies) in a 25 μl final volume. Primers are shown in Table 1.

**Table 1:** Primers for quantitative real-time PCR.

**Gene**	**Forward primer**	**Reverse primer**
**RAT**		
Hepcidin	5’-ctgcctgtctcctgcttctc-3’	5’-gttggtgtctcgcttccttc-3’
Ferritin H	5’-gctgaatgcaatggagtgtg -3’	5’-tcttgcgtaagttggtcacg -3’
Ferritin L	5’-agtggggtaaaaccctggag-3’	5’-agagtgaggcgctcaaagag-3’
TfR-1	5’-tgaaactggctgcagatgag-3’	5’-ttctgacttgtccgcctctt-3’
TNFα	5’-actcccagaaaagcaagcaa-3’	5’-ggccatggaactgatgagag-3’
IL-6	5’-ccggagaggagacttcacag-3’	5’-acagtgcatcatcgctgttc-3’
SOD2	5’-tggccaagggagatgttaca-3’	5’-taaggcctgtggttccttgc-3’
HMOX1	5’-caagctctggggaaggcc-3’	5’-agtatcttgaaccaggctagca -3’
Fp-1	5’-ccctgctctggctgtaaaag-3’	5’-taggagacccatccatctcg-3’
IRP1	5’-tcaacagaagggcagacagtt-3’	5’-acctacaccccaaccaagaa-3’
IRP2	5’-tcaagatttcactggaataccg-3’	5’-aacaccgtctcaggttcagg-3’
beta-actin	5’-atggtgggtatgggtcagaa-3’	5’-ggggtgttgaaggtctcaaa-3’
**HUMAN**		
Hepcidin	5’-gaccagtggctctgtttt-3’	5’-cacatcccacactttgat-3’
Ferroportin	5’-ccaaagggattggattgt-3’	5’-aaataaagccacagccga-3’
TfR-1	5’-gaggagccaggagagga-3’	5’-acgccagactttgctga-3’
IRP1	5’-ctgcaggactttacgggtgt-3’	5’-tcacctggtggatgattcct-3’
Ferritin H	5’tcaagaaaccagactgtgatg-3’	5’-gtcacacaaatgggggtcat-3’
beta-actin	5’-ggcatcctcaccctgaagta-3’	5’ggggtgttgaaggtctcaaa-3’

Tmprss6, transmembrane protease serine 6; TfR-1, transferrin receptor; TfR-2, transferrin receptor 2; TNFα, tumor necrosis factor alpha; BMP, bone morphogenetic protein, IRP, iron responsive protein.

### Western Blot Analysis

Tissue samples (15–20 μg) were homogenized in 1 mL of Trizol reagent and proteins were isolated from the phenol-ethanol supernatant obtained after DNA precipitation with isopropyl alcohol. After three washes in a solution containing 0.3M guanidine hydrochloride the protein pellet was washed in ethanol and then dissolved in 1% SDS. Cells were lysed in RIPA buffer containing 1 mmol/L Na-orthovanadate, 200 mmol/L phenylmethyl sulfonyl fluoride, and 0.02 μg/μL aprotinin.

Equal amounts of proteins (50 μg) were separated by sodium dodecyl sulfate polyacrylamide gel electrophoresis and transferred electrophoretically to nitrocellulose membrane (BioRad, Hercules, CA). Membranes were incubated with polyclonal rabbit Ferritin H antibody (sc-25617, Santa Cruz Biotechnology, Santa Cruz, CA), polyclonal rabbit Ferritin L antibody (sc-25616, Santa Cruz Biotechnology), polyclonal mouse transferrin receptor-1 (TfR-1) antibody (13–6800, Zymed, Life Technologies), polyclonal mouse Fp-1 antibody (MTP11-A, Alpha Diagnostic, San Antonio, USA), polyclonal rabbit IRP1 antibody (NBP1-87677, Novus Biologicals, Cambridge, UK), monoclonal rabbit AKT antibody (C73H10, Cell Signaling, Danvers, MA), monoclonal rabbit pThr308 AKT (C31E5E, Cell Signaling, Danvers, MA), polyclonal goat β-actin antibody (sc-1615, Santa Cruz Biotechnology).

### Iron regulated protein activity

Iron Regulated Protein (IRP) binding activity was measured by RNA band shift assay as previously described [26].

### Cells

Human HepG2 hepatocytes were grown in DMEM medium supplemented with 10% fetal calf serum (FCS), 1% Penicillin Streptomycin and 1% L-GLN (Life Technologies) in a humidified atmosphere with 5% CO_2_ at 37°C. Viability was assessed by Trypan blue exclusion dye test. In order to evaluate the effect of increased exposure to fatty acids on iron metabolism, cells were treated with palmitic acid (PA 0.66 mM: saturated, C16:0) plus oleic acid (OA 1 mM: n-9 mono-unsaturated, C18:1, cis) 24 hours before the experiments. To assess the effect of specific types of fatty acids on iron metabolism, HepG2 cells were also treated for 24 hours with the following: linolenic acid (LnA 0.025 mM; n-3 unsaturated, 18:2), stearic acid (SA 0.025 mM: saturated, C18:0), elaidic acid (EA; 0.1 mM: n-9 mono-unsaturated, 18:1, trans), lauric acid (LA 0.2 mM: saturated medium-chain, C12:0). Iron was supplemented for 24 hours as ferric ammonium citrate (FAC) 150 μM [27]. Intracellular iron concentration was measured by atomic absorption spectrometry [25]. For lipid visualization and quantification through Oil Red O staining, after being washed 3x with PBS, cells were fixed with 10% formalin, stained with 0.7% Oil Red O solution (Sigma, St. Louis, MO) and absorbance was then read at 540 nm.

### IRP1 gene silencing

HepG2 cells were transfected with siRNA against human IRP1 (Life Technologies, Carlsbad, CA). Transfection was carried out by using TurboFect transfection reagent (Thermo Scientific, Waltham, MA, USA) and 5 pmol/μl siRNA per well, according to the manufacturer’s instructions. Negative control siRNA was used as control for the effect of RNA interference. Silencing was monitored at mRNA levels.

### Patients

We considered 46 Italian unrelated patients with NAFLD who underwent percutaneous liver biopsy because of persistently abnormal liver enzymes/serum ferritin or long-lasting history of steatosis associated with severe metabolic abnormalities. These included 23 patients with simple uncomplicated steatosis (SS) alone and 23 with nonalcoholic steatohepatitis (NASH). Ten subjects who underwent liver biopsy because of suspected NAFLD but without histological abnormalities were included as controls (NL: normal liver). Other causes of liver disease were excluded, including increased alcohol intake (<30/20 g/day for males/females). Demographic and clinical features are shown in Table 2. Informed written consent was obtained from each patient and control subject. The study was approved by the Ethical Committee of the Fondazione Ca’ Granda IRCCS of Milan and conforms to the principles of the Declaration of Helsinki.

**Table 2:** Clinical features of subjects with liver biopsy evaluated in the study.

	**Italian NAFLD**	**Controls**
Number	46	10
Sex M (%)	24 (52)	8 (18)
Age years	48±10	54.4±12
BMI (Kg/m^2^)	32.2±7.3	26.7±3.5
HDL cholesterol (mg/dl)	55±22	51±15
Triglycerides (mg/dl)	136±45	127±61.9
Glucose (mg/dl)	97±27	93.1±16.2
HOMA-IR	4±2.4	4.2±3.3
ALT (UI/ml)	44±31	38.1±24
Serum iron (μg/dl)	118±44	124±30
Ferritin (ng/ml)	689±457	240±186
TS (%)	39±17	47±8
TIBC (μg/dl)	302±50.2	261±20
NASH (%)	26 (50)	0

BMI, body mass index; HDL, high density lipoprotein; ALT, alanine transaminase; TS, transferring saturation; TIBC, total iron binding capacity; HOMA-IR, Homeostasis Model of Assessment-Insulin Resistance; NASH, nonalcoholic steatohepatitis.

### Histological assessment

Tissue sections were stained with hematoxylin-eosin, impregnated with silver for the reticulin framework, and stained with trichrome for collagen and Perls’ Prussian blue for iron. One expert pathologist unaware of clinical and genetic data (M.M.) reviewed all biopsies for fibrosis stage. The presence of NASH was assessed according to the NAFLD clinical research network criteria [28], and the presence and severity of iron stores graded according to Scheuer [29].

### Statistical analysis

Data are expressed as means ± SD. Statistical analysis was assessed by Anova or repeated measures Anova, when appropriate. Differences were considered statistically significant at p<0.05.

## Results

### HFD leads to mild steatosis and hepatic iron accumulation in rats

**Figure 1:**
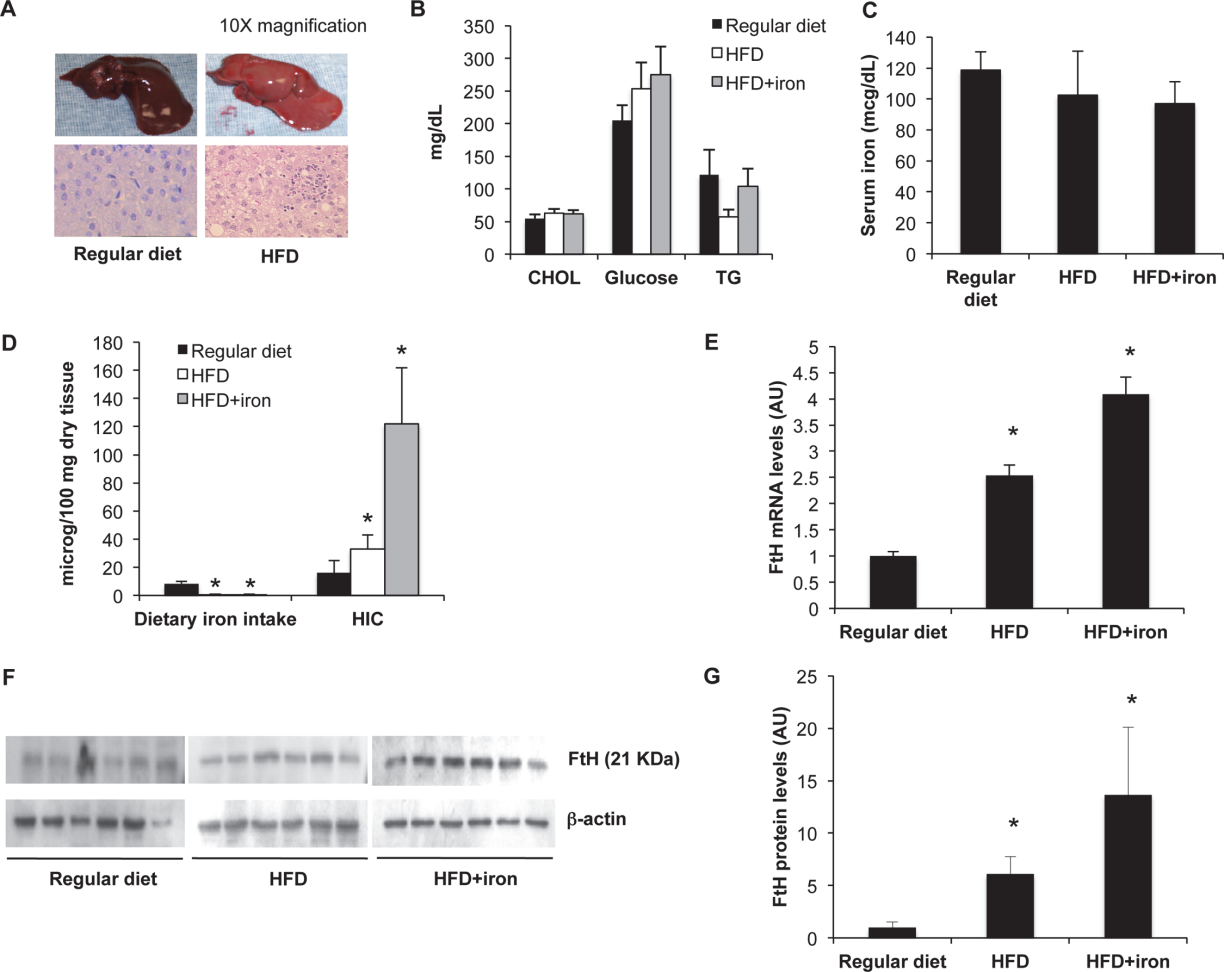
HFD determines mild steatosis and hepatic iron accumulation with increased ferritin. Rats were fed for 12 weeks with standard chow (n = 6), or high fat diet (HFD, n = 6), or HFD plus s.c. iron administration at week 8 (250 mg iron sulfate, n = 6). A) Representative images of hematoxylin-eosin staining of liver section with magnification of 10X as indicated. B) Concentrations of serum cholesterol, triglyceride and glucose. C) Serum iron levels. D) Dietary iron intake and hepatic iron concentration (HIC) measured by atomic absorption spectrometry. E) Ferritin H mRNA levels in HFD and HFD+iron rats compared to controls. Gene expression was evaluated by qRT-PCR. F) Ferritin H protein levels evaluated by Western Blotting. G) Densitometric analysis of ferritin H protein levels; β-actin is shown as the loading control. The figure is representative of results obtained in 6 animals per group, replicated in two independent experiments. Values are expressed as means±SD. AU, arbitrary units. *p<0.05 vs controls.

Rats fed HFD developed mild steatosis, which was not detected under regular diet. Macroscopical and histological evaluation of liver parenchyma is shown in Fig. 1A. Glucose levels, as well as total cholesterol and triglycerides were not significantly affected by HFD (Fig. 1B). ALT levels were not different across treatment groups (23±5 IU/ml regular diet, 27±8 HFD, 30±7 HFD+iron). Body weight, insulin levels and liver phosphorylation status of AKT, a key molecule involved in insulin signaling, were not significantly influenced by HFD (S2 Fig.).

Average dietary iron intake, based on weekly food intake and dietary iron content measured by atomic absorption spectrometry, was 10-fold lower in HFD treated rats compared to controls (0.74±0.3 *vs* 8.5±1.5 μg/week, p<0.0001; Fig 1D). However, serum iron did not differ across treatment groups (Fig. 1C). Remarkably, HIC was about 50% higher in HFD rats than in rats fed regular diet, (Fig. 1D), whereas as expected iron supplementation resulted in a further increase in HIC (16±9 μg/100 mg dry tissue in controls, 33±10 in HFD, 122±40 in HFD+iron, p = 0.01). Iron staining showed mild predominantly parenchymal iron depots in the liver of HFD rats (S1 Fig.).

In response to increased hepatic iron, mRNA levels of the iron storage protein H ferritin were increased in HFD and HFD+iron groups (P<0.05; Fig. 1E), which was paralleled by an increase in H ferritin protein levels in HFD rats compared to controls (p<0.05; Fig. 1F–G). mRNA levels of L ferritin were also increased in HFD rats compared to controls (S4A–B Fig.).

### HFD influences hepatic iron metabolism in rats

**Figure 2:**
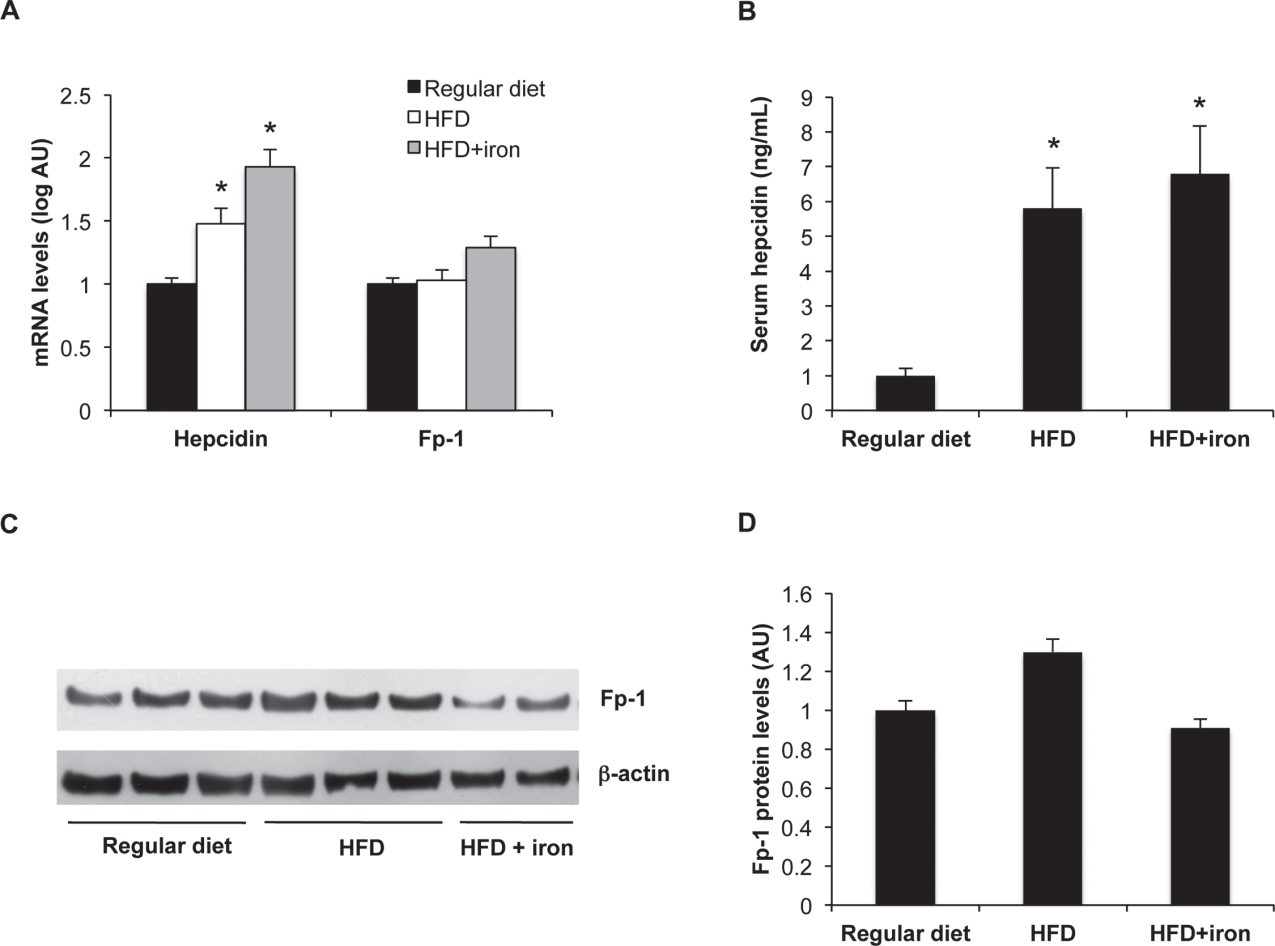
Effect of HFD on hepatic iron metabolism genes. A) Hepatic hepcidin and Fp-1 mRNA levels. Gene expression was evaluated by qt-PCR. B) Serum hepcidin levels were evaluated by an EIA kit. C) Hepatic Fp-1 protein levels evaluated by Western Blotting. D) Densitometric analysis of ferroportin protein levels; β-actin is shown as the loading control. The figure is representative of results obtained in 6 animals per group in two independent experiments. Values are expressed as means±SD. AU, arbitrary units. *p<0.05 vs controls.

To investigate the mechanisms underlying hepatic iron accumulation in HFD, we next analyzed the effect of HFD diet on the expression of hepcidin and Fp-1. In line with increased hepatic iron, hepcidin mRNA levels were higher in HFD rats compared to controls, and further increased by iron supplementation (1.1±0.3 controls, 1.7±0.4 HFD, 2.2±0.6 HFD+iron, p<0.05 for HFD and HFD+iron vs. regular diet; Fig. 2A). Accordingly, serum hepcidin levels were higher in HFD rats compared to controls (p<0.05; Fig. 2B). On the other hand, hepatic Fp-1 mRNA and protein levels were not affected by HFD (Fig. 2A, C–D).

As regard to inflammation, TNFα was not induced, whereas IL-6 mRNA levels were induced only when HFD was supplemented with iron (S3A Fig.; p<0.05). Concerning oxidative stress, manganese superoxide dismutase (SOD2) was not induced by HFD and also heme oxygenase-1 (HMOX1) mRNA levels were not significantly higher in HFD rats vs. controls (S3B Fig.).

Thus, HFD diet recapitulated the typical features of DIOS, including increased ferritin with normal serum iron, increased hepatic iron stores, and concomitant up-regulation of hepcidin, associated with mild hepatic steatosis.

### HFD induces Transferrin receptor upregulation in rat liver

Since hepcidin upregulation during HFD would be predicted to contrast iron absorption, but Fp-1 protein levels were not downregulated, hepatic iron accumulation in HFD remained unexplained. Therefore, we next evaluated the effect of HFD diet on Transferrin receptor-1 (TfR-1), the physiological mediator of iron uptake from plasma. TfR-1 is usually downregulated by iron stores through reduced binding of iron regulatory proteins (IRPs) that stabilize the messenger by binding to iron regulatory elements (IRE) localized in the 3’ untranslated region of mRNA [26].

**Figure 3:**
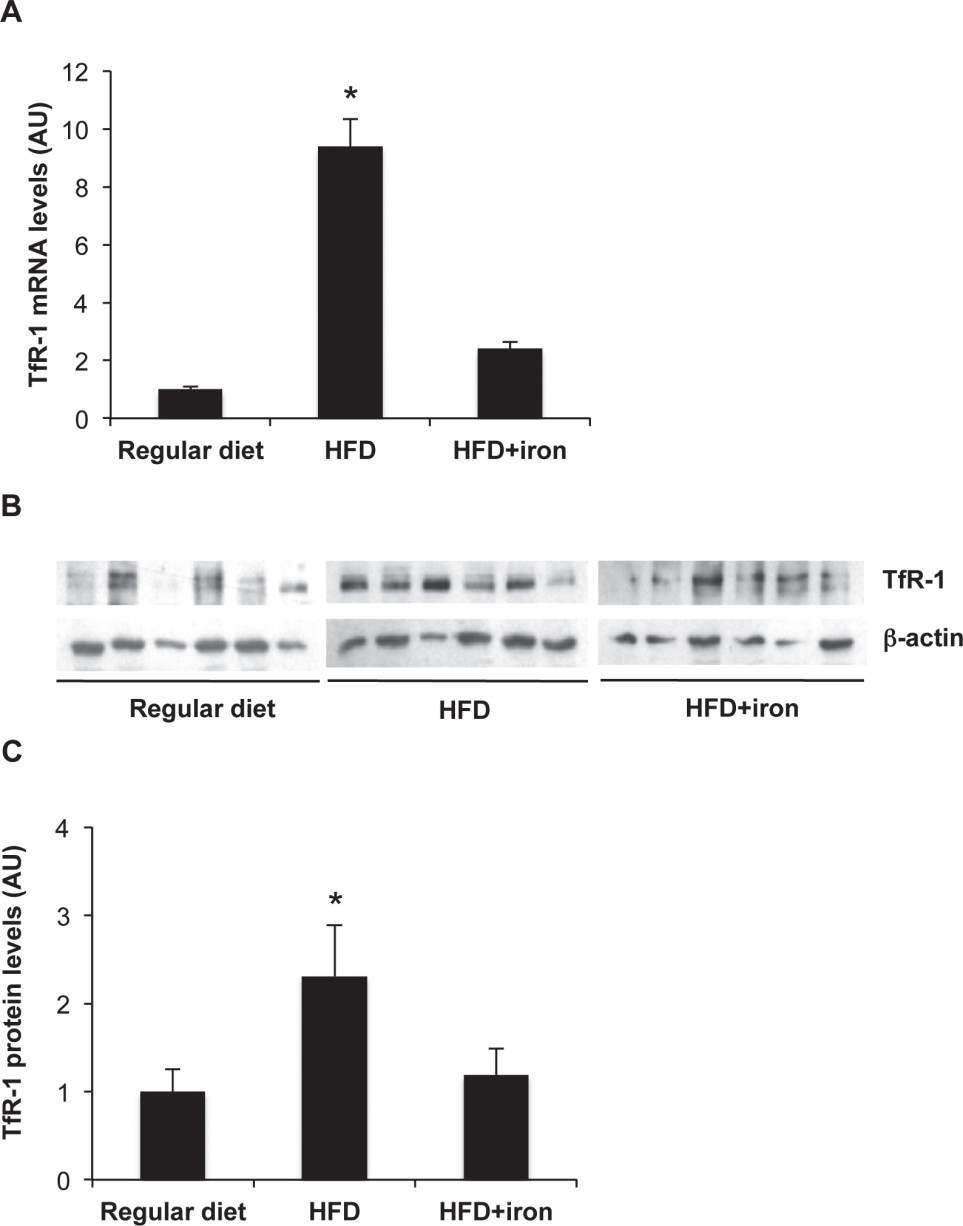
Effect of HFD on TfR-1 levels. A) Hepatic TfR-1 mRNA levels in HFD and HFD+iron rats compared to controls. Gene expression was evaluated by qRT-PCR. B) Hepatic TfR-1 protein levels evaluated by Western Blotting. C) Densitometric analysis of TfR-1 protein levels; β-actin is shown as the loading control. The figure is representative of results obtained in 6 animals per group in two independent experiments. Values are expressed as means±SD. AU, arbitrary units. *p<0.05 vs. controls.

Surprisingly, both hepatic mRNA and protein levels of TfR-1 were upregulated by HFD (2.3-fold, p<0.05), whereas TfR-1 levels were downregulated to the levels observed in regular diet, albeit to a degree not proportional to iron stores, by iron supplementation (Fig. 3A–C).

**Figure 4:**
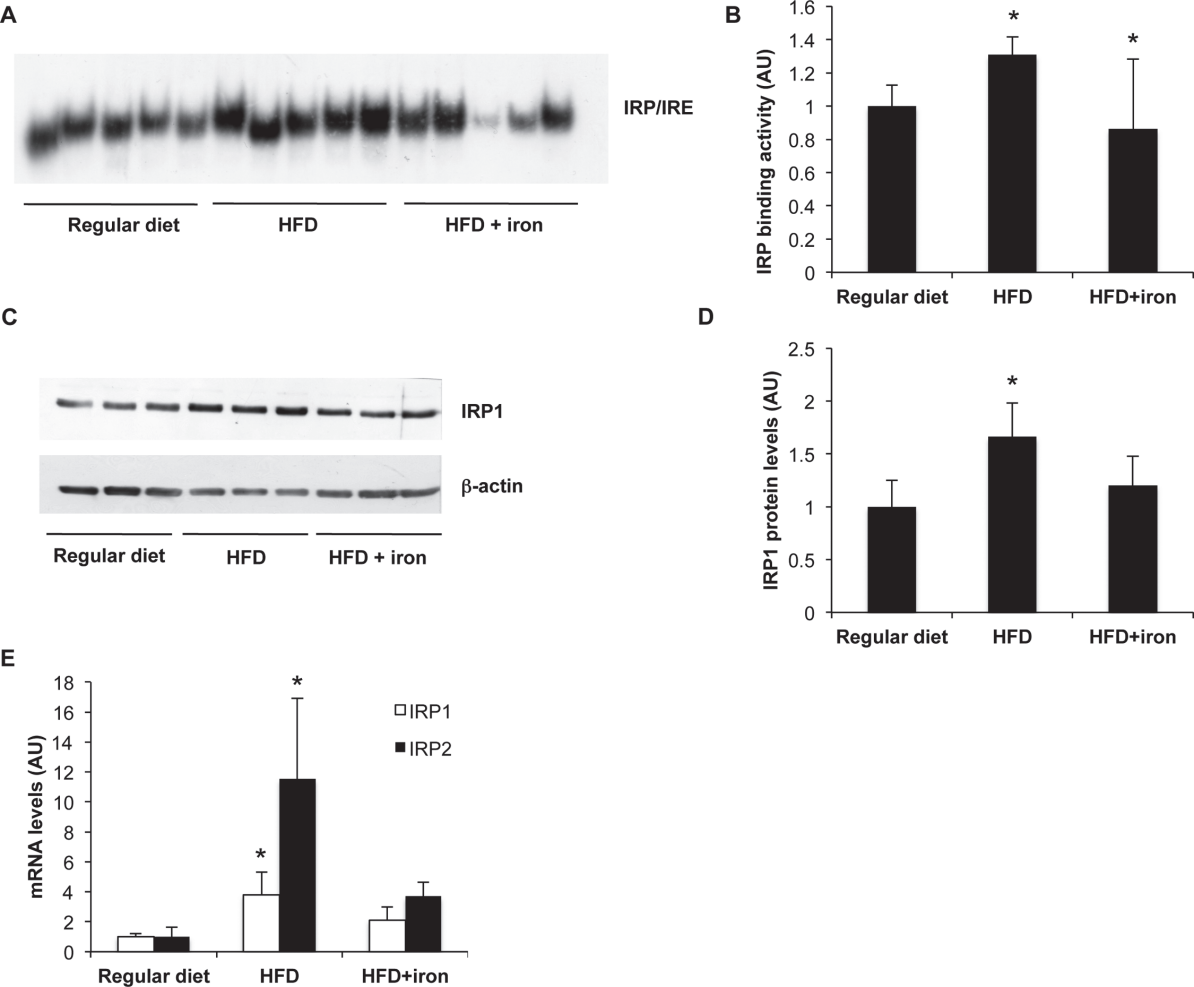
Effect of HFD on IRP1. A) Total IRP1 activity was measured by RNA band shift assay. B) Densitometric analysis of IRP1 activity. C) Hepatic IRP1 protein levels evaluated by Western Blotting. C) Densitometric analysis of IRP1 protein levels; β-actin is shown as the loading control. D). Effect of HFD on IRP1 and IRP2 mRNA levels. Gene expression was evaluated by qRT-PCR. The figure is representative of results obtained in 6 animals per group in two independent experiments. Values are expressed as means±SD. AU, arbitrary units. *p<0.05 vs controls.

To investigate the mechanism underlying TfR-1 upregulation by HFD, we then evaluated hepatic IRP activity and expression. The IRE binding activity of IRP was higher in HFD rats than in rats fed regular diet (p = 0.003), and HFD supplemented with iron (p<0.05; Fig. 4A–B). IRP binding activity depends both on IRP1/IRP2 levels and on intracellular free iron availability [30]. Increased IRP activity in rats fed HFD was proportional to increased IRP1 protein levels (p = 0.002 HFD vs. regular diet; Fig. 4C–D), reflecting increased IRP1 mRNA levels during HFD compared to control diet (Fig. 4E). IRP2 mRNA levels were also increased in HFD rats compared to controls (Fig. 4E).

### Fatty acids upregulate TfR-1 and favor iron accumulation in hepatoma cells

In order to investigate whether specific dietary FFA directly interferes with iron metabolism in hepatocytes, we challenged hepatoma HepG2 cells with physiological concentrations of palmitic acid (PA, 0.66 mM, the most common dietary saturated fatty acid) plus oleic acid (OA, 1 mM, the most common dietary monounsaturated fatty acid) for 24 hours.

**Figure 5:**
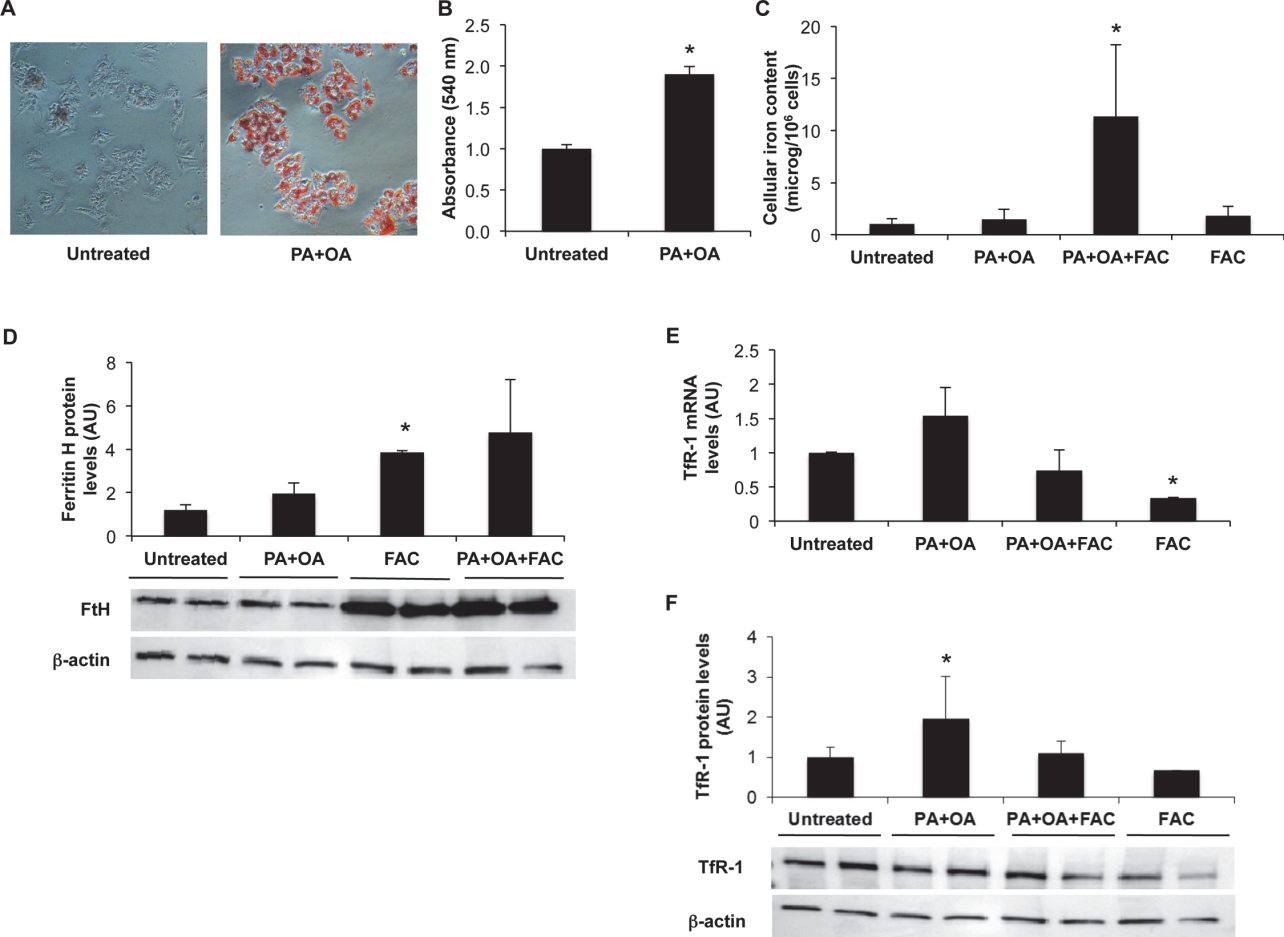
Effect of FFA treatment on iron metabolism in HepG2 hepatocytes. HepG2 hepatocytes were incubated with PA (0.66 mM) + OA (1 mM) or with FAC (150 μM) alone or in combination with FFA for 24 hours. A) Cells after Red Oil O staining. Intensity of red color is proportional to lipid accumulation. B) Absorbance of Red Oil O (540 nm). C) Intracellular iron concentration was measured by atomic absorption spectrometry. D) Ferritin H protein levels were evaluated by Western Blotting (lower part). Densitometric analysis of ferritin H protein levels is shown in the upper part of the figure; β-actin is shown as the loading control. E) TfR-1 mRNA levels evaluated by qRT-PCR. F) TfR-1 protein levels were evaluated by Western Blotting (lower part). Densitometric analysis of TfR-1 protein levels is shown in the upper part of the figure; β-actin is shown as the loading control. Results are mean values of three independent experiments, each experimental condition was evaluated in triplicate. Values are expressed as means±SD. AU, arbitrary units. *p<0.05 vs. controls.

To verify the effect of FFA addition, Oil Red O staining was performed, and lipid content quantification indicated that HepG2 cells treated with PA+OA had almost twice fat stored (1.9 times, p<0.05) compared to untreated cells (Fig. 5A–B).

Intracellular iron concentration was increased in HepG2 cells treated with fatty acids (PA+OA) plus iron, whereas fatty acids, and remarkably also FAC alone, did not increase cellular iron content (p<0.05; Fig. 5C). In line with increased iron content, H ferritin protein levels increased in cells treated with fatty acids plus FAC compared to untreated cells.

Conversely, FAC increased H ferritin protein levels without significantly affecting intracellular iron content, suggesting reduced ability of ferritin to store iron under these experimental conditions (Fig. 5D).

The mRNA and protein expression of TfR-1 were increased in cells treated with a combination of fatty acids (Fig. 5E–F). As expected, TfR-1 was downregulated in the presence of FAC supplementation.

**Figure 6:**
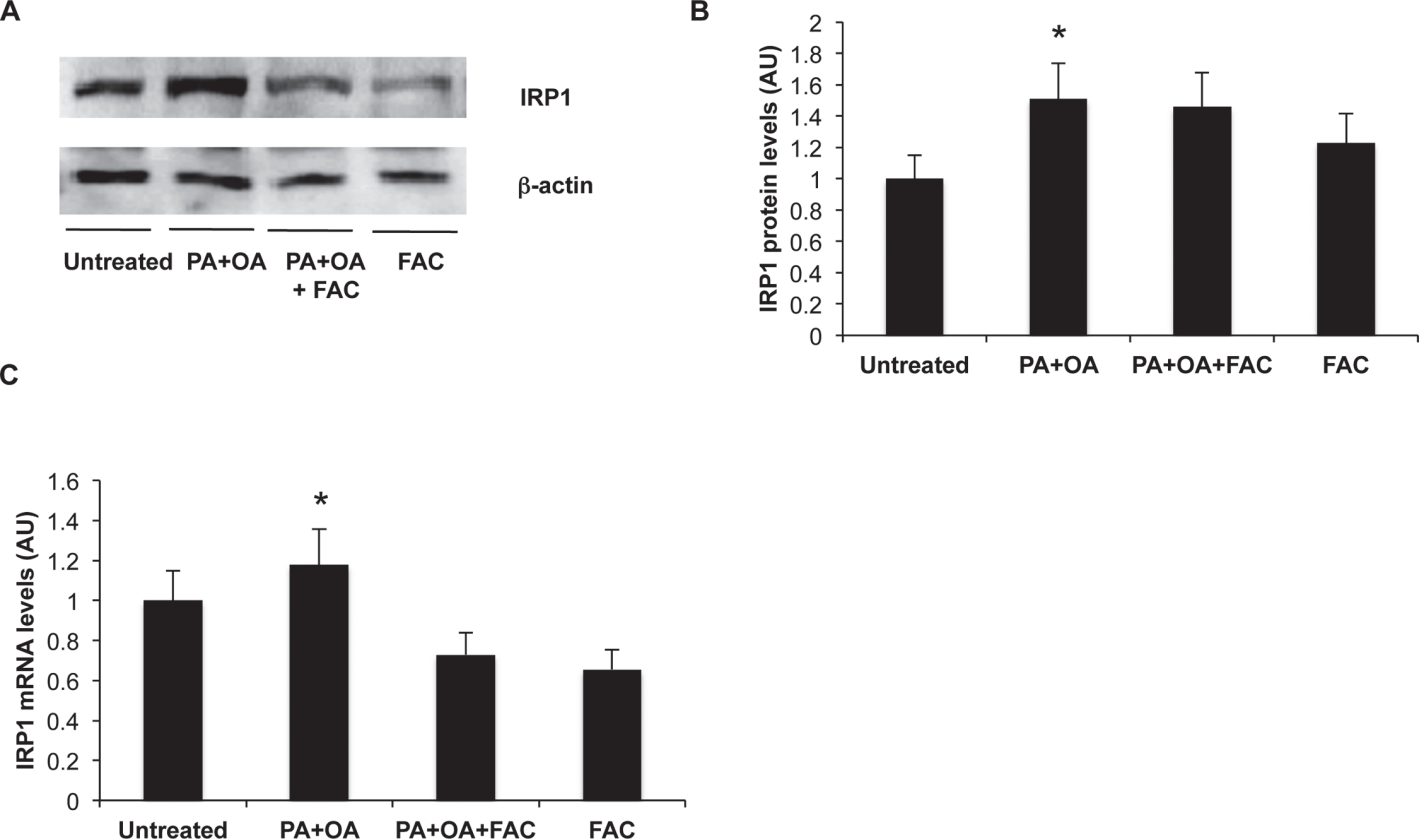
Effect of fatty acids on IRP1 expression in HepG2 hepatocytes. A) IRP1 protein levels were evaluated by Western Blotting. B) Densitometric analysis of IRP1 protein levels: β-actin is shown as the loading control. C) IRP1 mRNA levels evaluated by qRT-PCR. Results are mean values of three independent experiments, each experimental condition was evaluated in triplicate. Values are expressed as means±SD. AU, arbitrary units. *p<0.05 vs. controls.

In line with the findings obtained *in vivo*, IRP1 protein and mRNA levels were increased in cells supplemented with FFA compared to vehicle alone (Fig. 6A–C). In addition, IRP binding activity was not upregulated in HepG2 exposed to FFA and it paradoxically increased in HepG2 treated with FFA plus FAC despite increased intracellular iron accumulation (S5A–B Fig.).

Moreover, intracellular iron concentration was significantly increased in HepG2 cells treated with different types of fatty acids plus FAC compared to untreated cells or to cells incubated with FFA alone (S6A Fig.; p<0.05). We also observed an upregulation of TfR-1 mRNA and protein levels in cells treated with other types of FFA (S6B–D Fig.).

### Effect of IRP1 silencing on TfR-1 induction and facilitation of iron uptake following exposure to fatty acids

**Figure 7:**
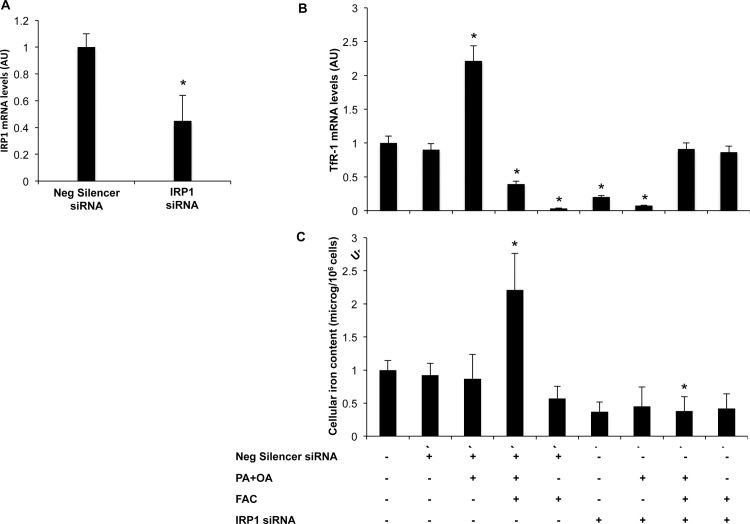
IRP1 gene silencing in HepG2 hepatocytes. A) IRP1 siRNA treatment of HepG2 cells reduced IRP1 mRNA levels compared to control cells (about 60%). B) TfR-1 mRNA levels evaluated by qRT-PCR in HepG2 treated with IRP1 siRNA or Negative silencer siRNA. C) Intracellular iron concentration was measured by atomic absorption spectrometry in HepG2 cells treated with IRP1 siRNA or Negative silencer siRNA. Results are mean values of three independent experiments, each experimental condition was evaluated in triplicate. Values are expressed as means±SD. AU, arbitrary units. *p<0.05 vs. untreated cells plus transfection reagent (lipofectamine).

To determine whether IRP1 is involved in the upreguation of TfR-1 and facilitation of hepatocellular iron accumulation by FFA, we next silenced IRP1 in HepG2 cells using a siRNA approach. IRP1 silencing resulted in a significant 60% decrease in IRP1 in mRNA levels (p<0.05; Fig. 7A). IRP1 silencing abrogated induction of TfR-1 following exposure to FFA (Fig. 7B), indicating that TfR-1 upregulation by fatty acids requires IRP1. Moreover, IRP1 silencing completely inhibited intracellular iron accumulation induced by exposure to fatty acids plus iron (Fig. 7C).

These data suggest that TfR-1 upregulation and facilitation of intracellular iron uptake induced by fatty acids are dependent on the IRP1 pathway.

### Hepatic iron accumulation is associated with TfR-1 upregulation in DIOS

To assess the translational relevance of these findings, we finally evaluated the expression of iron genes in patients with fatty liver with and without hepatic iron accumulation.

**Figure 8:**
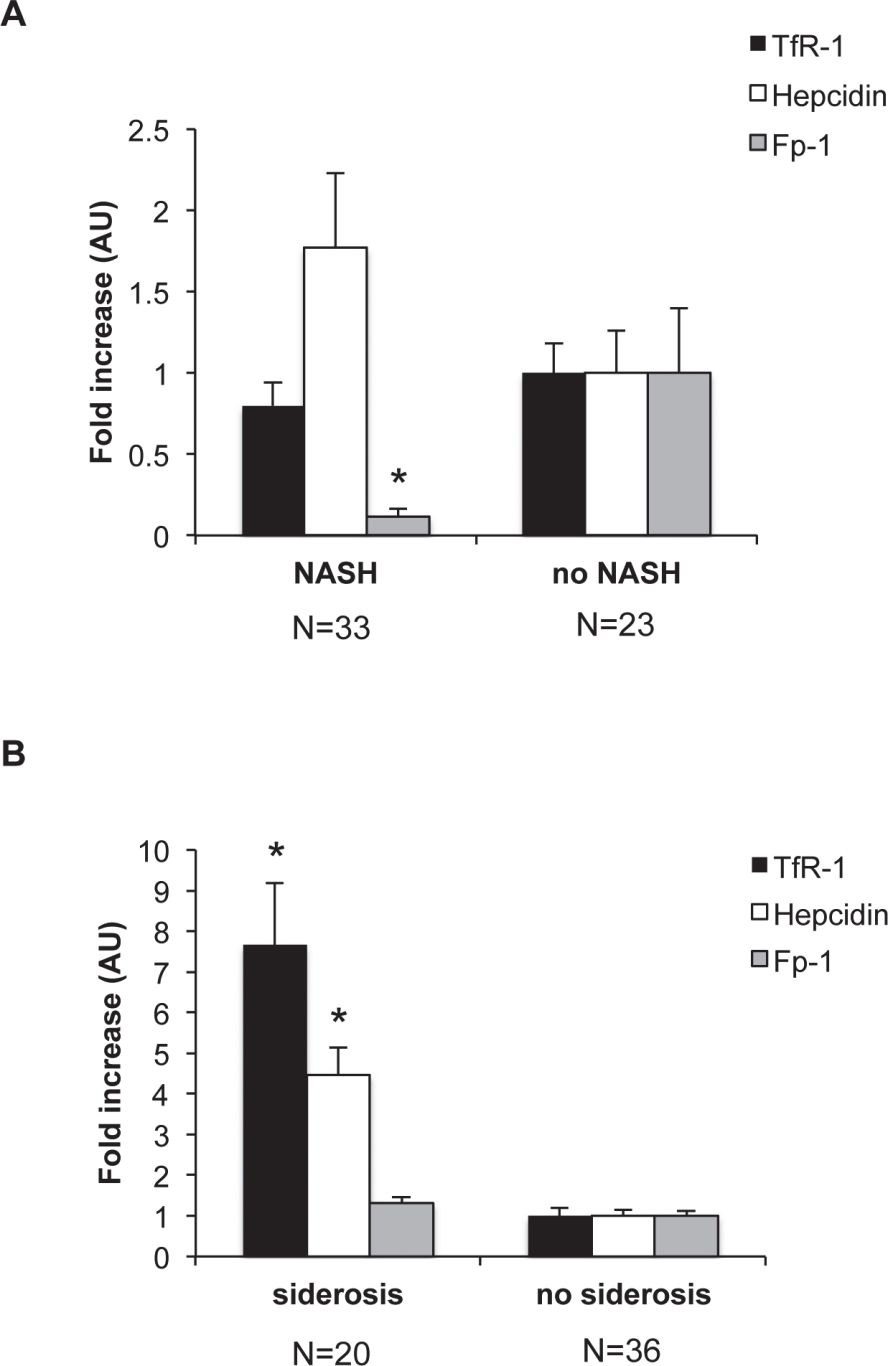
Association between steatosis and expression of iron genes in patients with fatty liver and healthy controls. A) Expression of hepatic hepcidin, Fp-1, TfR-1 mRNA levels according to liver damage (normal liver and simple steatosis, vs. nonalcoholic steatohepatitis: NASH), evaluated by qRT-PCR. B) Relative expression of TfR-1, Fp-1, and hepcidin according to the presence of siderosis. Values are expressed as means±SD. AU, arbitrary units. *p<0.05 vs. controls.

As previously reported [22], we observed decreased Fp-1 mRNA expression in patients with steatosis and inflammation (NASH) than in those without NASH (NL and SS; Fig. 7A). Conversely, hepcidin expression was increased in NASH vs. no NASH (Fig 8A), whereas TfR-1 was not associated with liver fat content or inflammation.

We next analyzed the relationship between the expression of iron genes and hepatic iron accumulation. The presence of histologically detectable iron stores was associated with an increased expression of TfR-1 and hepcidin mRNA (Fig. 8B). In contrast, there was no association between the presence of hepatic siderosis and Fp-1 expression. These results suggest that deregulation of TfR-1 expression is specifically associated with hepatic iron accumulation, but not with fat accumulation or inflammation.

## Discussion

To investigate the mechanisms underlying DIOS, in this paper we evaluated the effect of FFA on hepatic iron metabolism. The main finding was that exposure of hepatocytes to FFA, leading to steatosis, was associated with a subversion of iron metabolism characterized by increased expression of the iron uptake protein TfR-1, and facilitation of iron storage.

First, in rats fed HFD we observed increased hepatic iron accumulation despite markedly lower dietary iron intake. This was associated with increased ferritin, normal serum iron, and preserved upregulation of hepcidin in the presence of mild hepatic steatosis, a consequence of increased FFA flux to the liver [31], recapitulating the typical features of DIOS [6,7]. Increased hepcidin expression after chronic iron loading confirms that hepcidin is not only regulated by plasma iron, but also by iron stores, and that a distinct regulatory mechanism that senses hepatic iron may modulate hepcidin response to chronic iron loading [32].

Facilitation of hepatic iron accumulation was not explained by decreased hepcidin nor by reduced expression of Fp-1, which was not downregulated by hepcidin possibly due to the balanced effect of inflammation, increased hepcidin and hepatocellular iron stores [22,33], or a possible state of hepcidin resistance in specific cell types. Therefore, we next looked at the regulation of the cellular iron uptake protein TfR-1.

HFD was associated with increased expression of TfR-1, which was only relatively blunted after additional iron supplementation. These data suggest that HFD directly induces TfR-1, which may be involved in the pathogenesis of DIOS via increased intracellular iron uptake. Increased levels of TfR-1 have previously been reported in alcoholic liver disease in correlation with hepatocellular iron overload, and ascribed to stabilization of IRPs following hepatic oxidative stress [34].

To investigate the mechanism leading to TfR-1 upregulation by HFD, we thus evaluated the activity and expression of IRP, major regulator of TfR-1 mRNA at post-transcriptional level [35]. We found upregulation of IRP activity in HFD rats, which was proportional to IRP1 protein, but also to higher IRP1 and IRP2 mRNA levels.

These findings are in line with those recently reported in a similar model in rats [36], where HFD was associated with iron accumulation and with upregulation of IRP1 and TfR-1. However, in the aforementioned model ferritin (which is commonly increased in NAFLD patients) was downregulated, whereas IRP1 induction was dependent on post-translational stabilization by oxidative stress [36]. Since others reported discrepant results on the effect of high fat on hepatic iron metabolism [37,38], more work analyzing the specific composition of different HFD models is required to clarify the observed discrepancies.

To better characterize the relationship between steatosis and iron metabolism, we next evaluated *in vitro* whether FFA directly interfere with iron uptake in HepG2 hepatocytes, characterized by preserved regulation of iron metabolism [39]. We confirmed that FFA led to increased hepatocellular lipid content and induction of TfR-1, associated with IRP1 upregulation.

Intracellular iron accumulation was detected in cells exposed to FFA plus iron salts compared to untreated cells and to FFA or iron alone, thereby suggesting that FFA exposure has a synergistic effect with increased extracellular availability on iron uptake. Therefore, FFA seem to directly perturb iron metabolism in hepatocytes probably through a mechanism encompassing induction of IRP1/TfR-1 thereby bypassing the negative feedback regulation of intracellular iron on TfR-1 mRNA stability via IRP1 inactivation [35].

Importantly, IRP1 silencing completely abrogated induction of TfR-1 and intracellular iron accumulation following exposure to FFA. These data suggest that TfR-1 upregulation and facilitation of intracellular iron uptake induced by fatty acids are dependent on the IRP1 pathway, and that upregulation of IRP1/TFR-1 has a causal role in facilitating iron accumulation following exposure to excess fatty acids.

Remarkably, it has recently been shown that ferritin contains FFA binding sites, whose occupancy modulates iron uptake and release. FFA binding by ferritin would enhance iron mineralization, decrease iron release and protect FA from oxidation [40]. Ferritin binding by increased cytoplasmic FFA would thus expect to lower intracellular free iron, thereby possibly explaining IRP1 and TfR-1 upregulation in the presence of increased hepatocellular iron uptake and ferritin observed in *in vivo* and *in vitro* experimental models.

To assess the translational relevance of these findings, we finally evaluated whether TfR-1 expression was associated with hepatic fat, inflammation, and iron stores in patients with fatty liver and controls. As previously reported [22], Fp-1 mRNA levels were decreased, whereas hepcidin was increased in patients with NASH. Conversely, TfR-1 was not associated with NASH, but was specifically associated with the presence of histologically detectable siderosis. In contrast, iron accumulation was not associated with inflammation or downregulation of Fp-1 expression, whereas in line with previous reports [6,7,21] and our experimental data, upregulation of hepcidin was preserved. These results suggest that in individuals with steatosis hepatic iron accumulation is counter-intuitively associated with TfR-1 induction, which thereby is possibly involved in the hepatic iron accumulation and dysregulation of iron metabolism of DIOS.

## Conclusions

In conclusion, both in experimental models *in vivo* in rats and *in vitro* in HepG2 hepatocytes, as well as in patients with DIOS, increased exposure to FFA seems to subvert hepatic iron metabolism, favoring the induction of an iron uptake program via IRP1 and TfR-1 despite hepatocellular iron accumulation.

## Supporting Information

S1 Fig Effect of HFD on hepatic iron accumulation. A) Representative images of liver section stained with Perls’ Prussian blue staining in rats were fed for 12 weeks with standard chow, or HFD, or HFD plus s.c. iron administration. The figure is representative of results obtained in 6 animals per group in two independent experiments. Original magnification: 10X.
(PPTX)

S2 Fig Effect of HFD on metabolic parameters and hepatic insulin signaling. A) Body weight of rats fed regular diet, HFD or HFD plus iron for 12 weeks. B) Serum insulin levels evaluated by ELISA. C) Hepatic pThr308AKT/AKT ratio evaluated by Western Blotting. D) Densitometric analysis of pThr308AKT/AKT ratio; β-actin is shown as the loading control. The figure is representative of results obtained in 6 animals per group in two independent experiments. Values are expressed as means±SD. AU, arbitrary units.
(PPTX)

S3 Fig Effect of HFD on hepatic inflammation. A) Hepatic mRNA levels of TNFα and IL-6. B) Hepatic mRNA levels of SOD2 and HMOX1. Gene expression was evaluated by qRT-PCR. The figure is representative of results obtained in 6 animals per group in two independent experiments. Values are expressed as means±SD. AU, arbitrary units. *p<0.05 vs. controls.
(PPTX)

S4 Fig Effect of HFD on hepatic expression of ferritin L. A) Ferritin L mRNA levels in HFD and HFD+iron rats compared to controls. Gene expression was evaluated by qRT-PCR. B) Ferritin L protein levels evaluated by Western Blotting (data not shown). Densitometric analysis of ferritin L protein levels; β-actin is shown as the loading control. The figure is representative of results obtained in 6 animals per group in two independent experiments. Values are expressed as means±SD. AU, arbitrary units. *p<0.05 vs. controls.
(PPTX)

S5 Fig Effect of FFA on IRP activity in HepG2 hepatocytes. A) Total IRP1 activity was measured by RNA band shift assay. B) Densitometric analysis of IRP1 activity. Results are mean values of three independent experiments, each experimental condition was evaluated in triplicate. Values are expressed as means±SD. AU, arbitrary units. *p<0.05 vs. controls.
(PPTX)

S6 Fig Effect of FFA treatment on iron metabolism in HepG2 hepatocytes. HepG2 hepatocytes were incubated with LnA (0.025 mM), SA (0.025 mM), EA (0.1 mM), LA (0.2 mM) or with FAC (150 μM) alone or in combination with FFA for 24 hours. A) Intracellular iron concentration was measured by atomic absorption spectrometry. B) TfR-1 mRNA levels evaluated by qRT-PCR. C) TfR-1 protein levels were evaluated by Western Blotting. β-actin is shown as the loading control. D) Densitometric analysis of TfR-1 protein levels. Results are mean values of three independent experiments, each experimental condition was evaluated in triplicate. Values are expressed as means±SD. AU, arbitrary units. *p<0.05 vs. controls (normalized for β-actin).
(PPTX)
