# Correction: High Fat Diet Subverts Hepatocellular Iron Uptake Determining Dysmetabolic Iron Overload

**DOI:** 10.1371/journal.pone.0120457

**Published:** 2015-03-16

**Authors:** The PLOS ONE Staff

There is an error in the Funding section. The publisher apologizes for the error. The correct funding information is as follows: This paper was supported by the following grants: Bando Giovani Ricercatori Ricerca Finalizzata 2007, Ministero della Salute e delle Politiche Sociali (Italian ministry of Health grant; GR-2007–683265) to LV. Paola Dongiovanni was financed by Italian fiscal contribution 5X1000–2010 devolved to Fondazione IRCCS Cà Granda Ospedale Maggiore Policlinico. The funders had no role in study design, data collection and analysis, decision to publish, or preparation of the manuscript.
