# Supplementary figures and images for: High Fat Diet Subverts Hepatocellular Iron Uptake Determining Dysmetabolic Iron Overload
Source: PLoS One. 2015 Feb 3;10(2):e0116855. doi: 10.1371/journal.pone.0116855 (PMC4315491; doi:10.1371/journal.pone.0116855)

## Slide 1
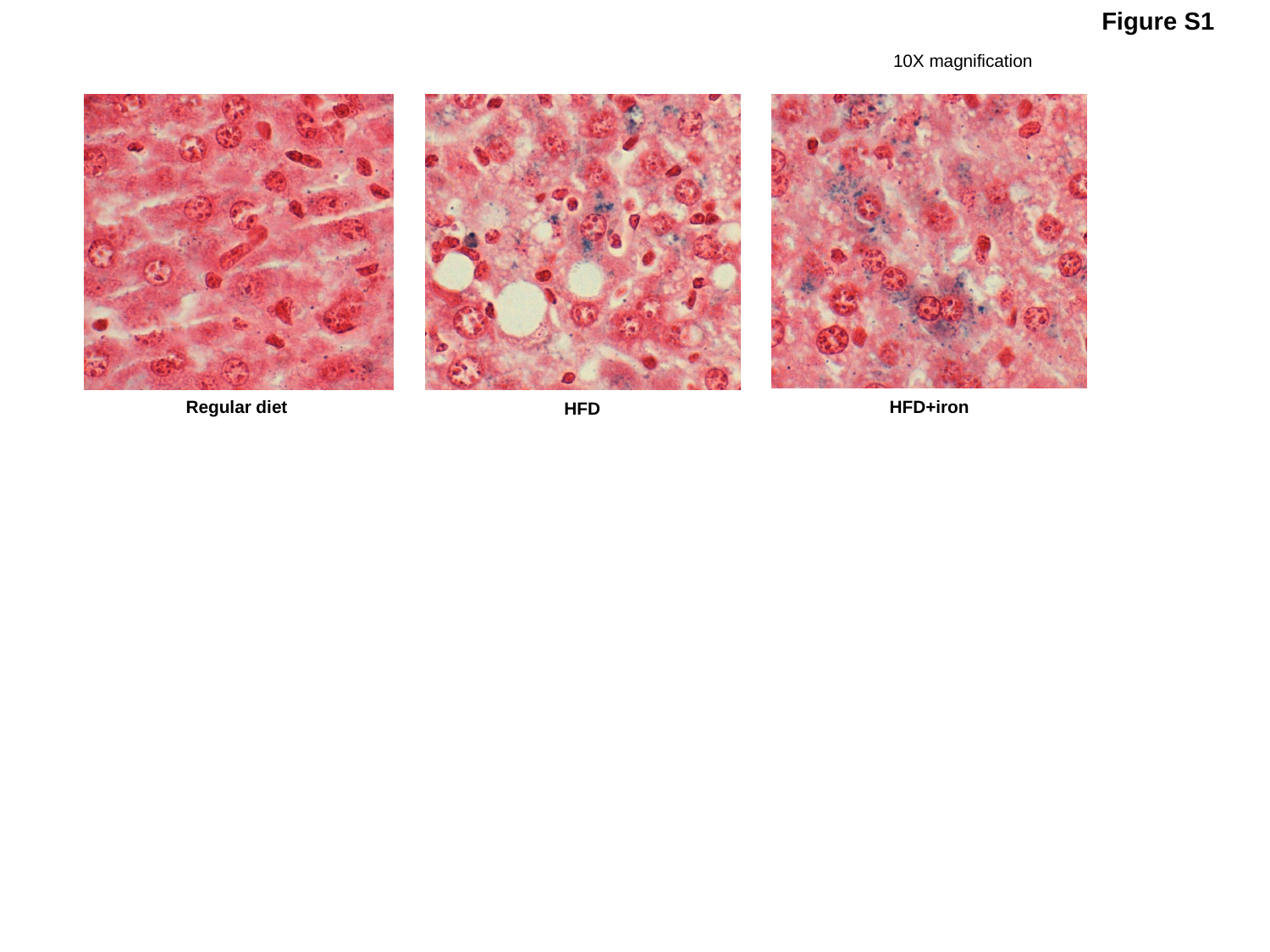

Figure S1
10X magnification
Regular diet
HFD+iron
HFD

Supplement: S1 Fig — A) Representative images of liver section stained with Perls’ Prussian blue staining in rats were fed for 12 weeks with standard chow, or HFD, or HFD plus s.c. iron administration. The figure is representative of results obtained in 6 animals per group in two independent experiments. Original magnification: 10X. (PPTX) [file pone.0116855.s001.pptx]

## Slide 1
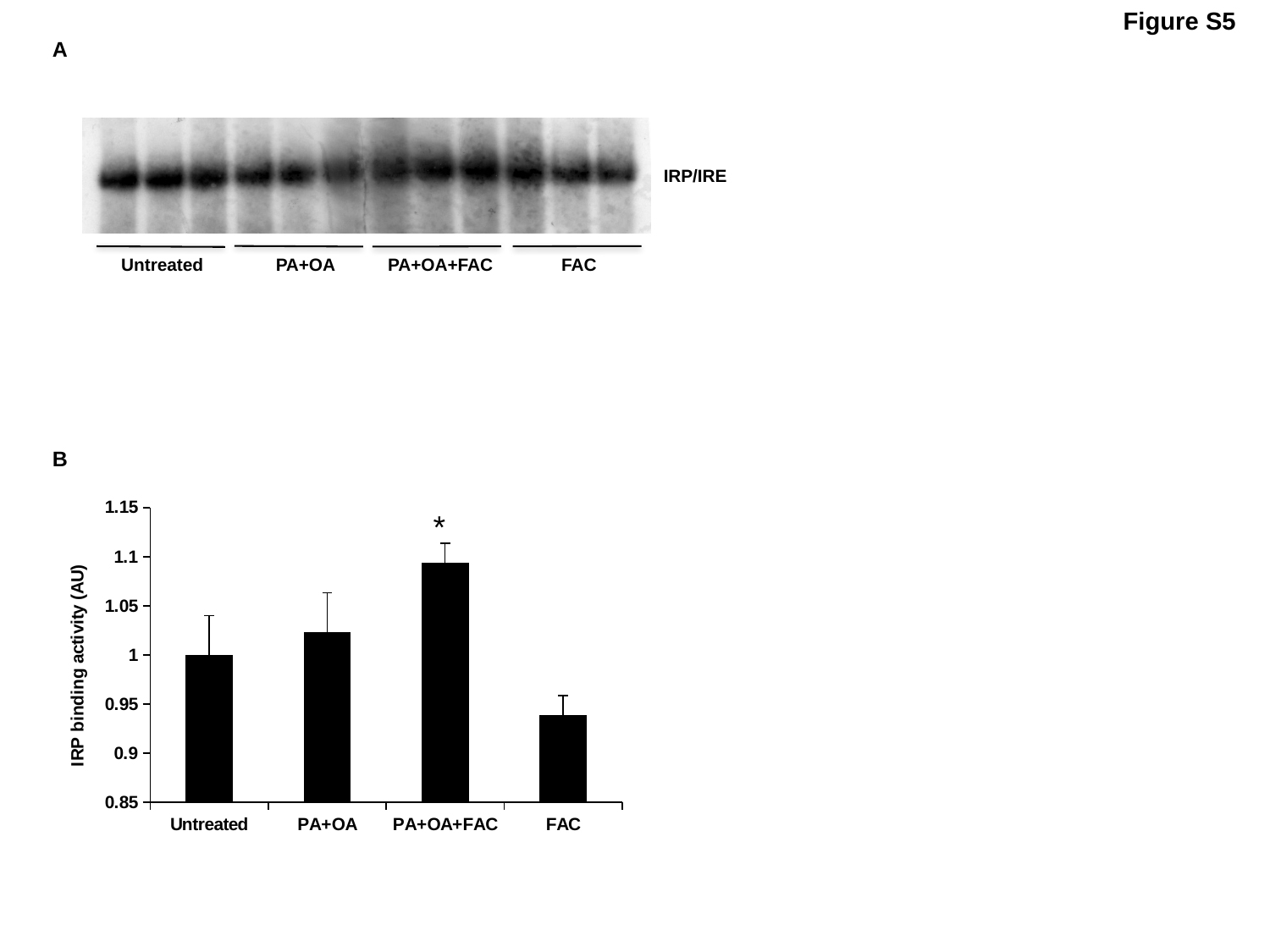

Figure S5
A
PA+OA
PA+OA+FAC
Untreated
FAC
IRP/IRE
B
### Chart
| Category | |
|---|---|
| Untreated | 1.0 |
| PA+OA | 1.023329798515376 |
| PA+OA+FAC | 1.093849416755037 |
| FAC | 0.938494167550371 |*

Supplement: S5 Fig — A) Total IRP1 activity was measured by RNA band shift assay. B) Densitometric analysis of IRP1 activity. Results are mean values of three independent experiments, each experimental condition was evaluated in triplicate. Values are expressed as means±SD. AU, arbitrary units. *p<0.05 vs. controls. (PPTX) [file pone.0116855.s005.pptx]
