# Supplementary material for: High Fat Diet Subverts Hepatocellular Iron Uptake Determining Dysmetabolic Iron Overload
Source: PLoS One. 2015 Feb 3;10(2):e0116855. doi: 10.1371/journal.pone.0116855 (PMC4315491; doi:10.1371/journal.pone.0116855)
Supplement: S2 Fig — A) Body weight of rats fed regular diet, HFD or HFD plus iron for 12 weeks. B) Serum insulin levels evaluated by ELISA. C) Hepatic pThr308AKT/AKT ratio evaluated by Western Blotting. D) Densitometric analysis of pThr308AKT/AKT ratio; β-actin is shown as the loading control. The figure is representative of results obtained in 6 animals per group in two independent experiments. Values are expressed as means±SD. AU, arbitrary units. (PPTX) [file pone.0116855.s002.pptx]

## Slide 1
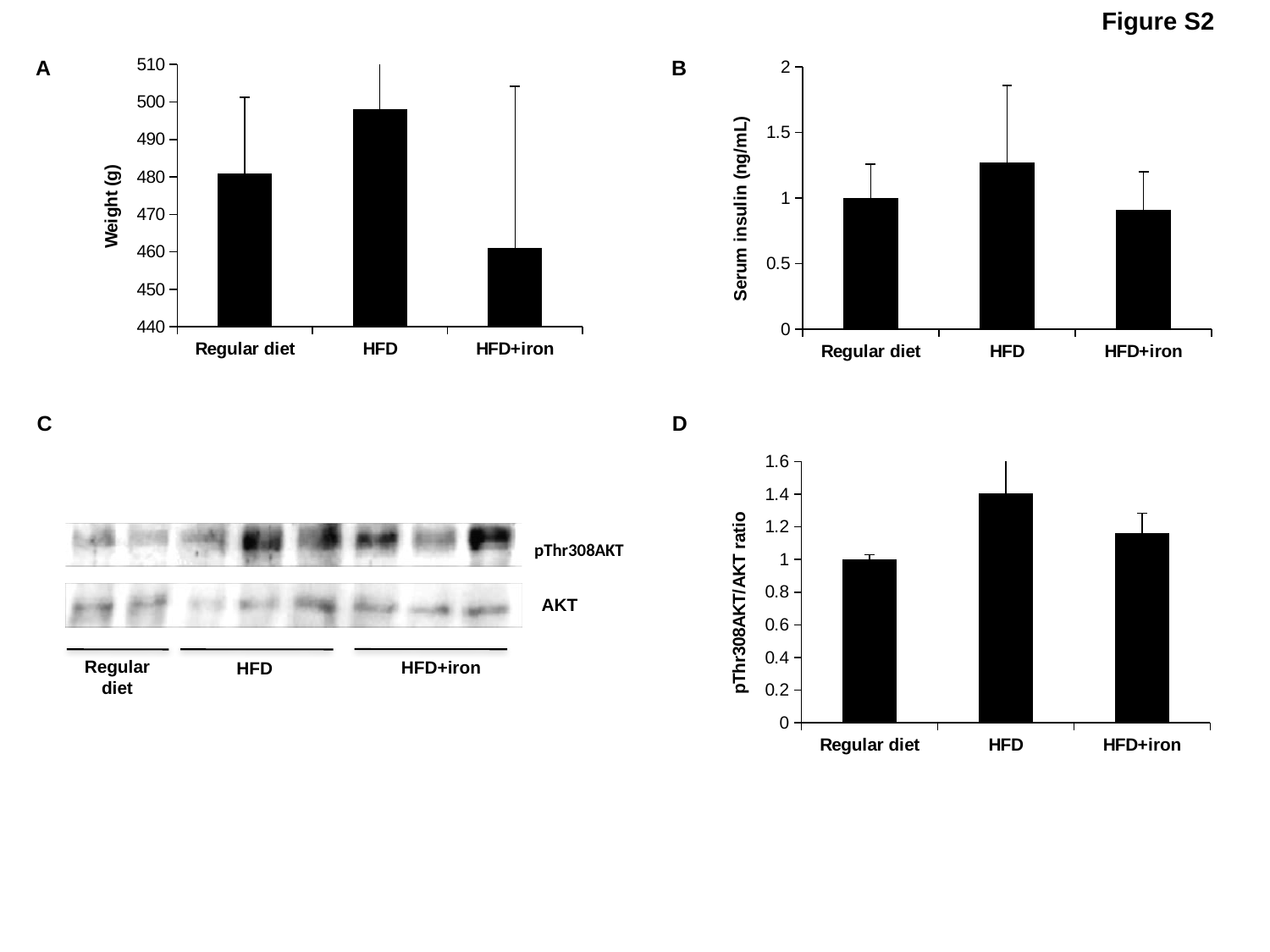

Figure S2
A
### Chart
| Category | Mean |
|---|---|
| Regular diet | 481.0 |
| HFD | 498.0 |
| HFD+iron | 461.0 |
### Chart
| Category | Insulin (ng/mL) |
|---|---|
| Regular diet | 1.0 |
| HFD | 1.27 |
| HFD+iron | 0.91 |B
C
D
### Chart
| Category | Mean |
|---|---|
| Regular diet | 1.0 |
| HFD | 1.405172413793103 |
| HFD+iron | 1.163793103448276 |
pThr308AKT
AKT
Regular diet
HFD+iron
HFD
