# Supplementary material for: High Fat Diet Subverts Hepatocellular Iron Uptake Determining Dysmetabolic Iron Overload
Source: PLoS One. 2015 Feb 3;10(2):e0116855. doi: 10.1371/journal.pone.0116855 (PMC4315491; doi:10.1371/journal.pone.0116855)
Supplement: S3 Fig — A) Hepatic mRNA levels of TNFα and IL-6. B) Hepatic mRNA levels of SOD2 and HMOX1. Gene expression was evaluated by qRT-PCR. The figure is representative of results obtained in 6 animals per group in two independent experiments. Values are expressed as means±SD. AU, arbitrary units. *p<0.05 vs. controls. (PPTX) [file pone.0116855.s003.pptx]

## Slide 1
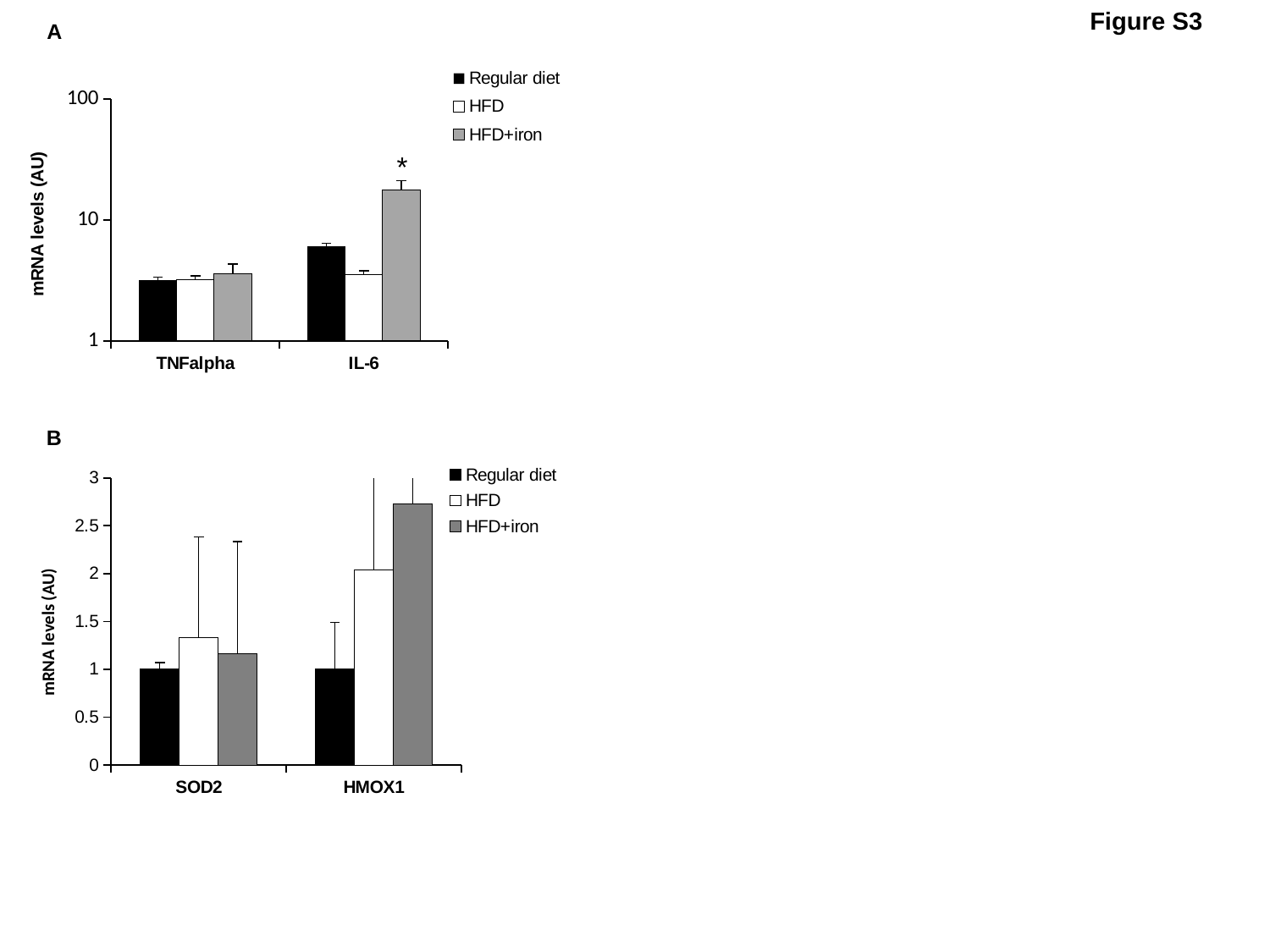

Figure S3
A
### Chart
| Category | Regular diet | HFD | HFD+iron |
|---|---|---|---|
| TNFalpha | 3.2 | 3.2 | 3.6 |
| IL-6 | 6.109999999999999 | 3.52 | 17.63 |*
B
### Chart
| Category | Regular diet | HFD | HFD+iron |
|---|---|---|---|
| SOD2 | 1.0 | 1.327777777777778 | 1.166666666666667 |
| HMOX1 | 1.0 | 2.04 | 2.73 |
