# Supplementary material for: High Fat Diet Subverts Hepatocellular Iron Uptake Determining Dysmetabolic Iron Overload
Source: PLoS One. 2015 Feb 3;10(2):e0116855. doi: 10.1371/journal.pone.0116855 (PMC4315491; doi:10.1371/journal.pone.0116855)
Supplement: S4 Fig — A) Ferritin L mRNA levels in HFD and HFD+iron rats compared to controls. Gene expression was evaluated by qRT-PCR. B) Ferritin L protein levels evaluated by Western Blotting (data not shown). Densitometric analysis of ferritin L protein levels; β-actin is shown as the loading control. The figure is representative of results obtained in 6 animals per group in two independent experiments. Values are expressed as means±SD. AU, arbitrary units. *p<0.05 vs. controls. (PPTX) [file pone.0116855.s004.pptx]

## Slide 1
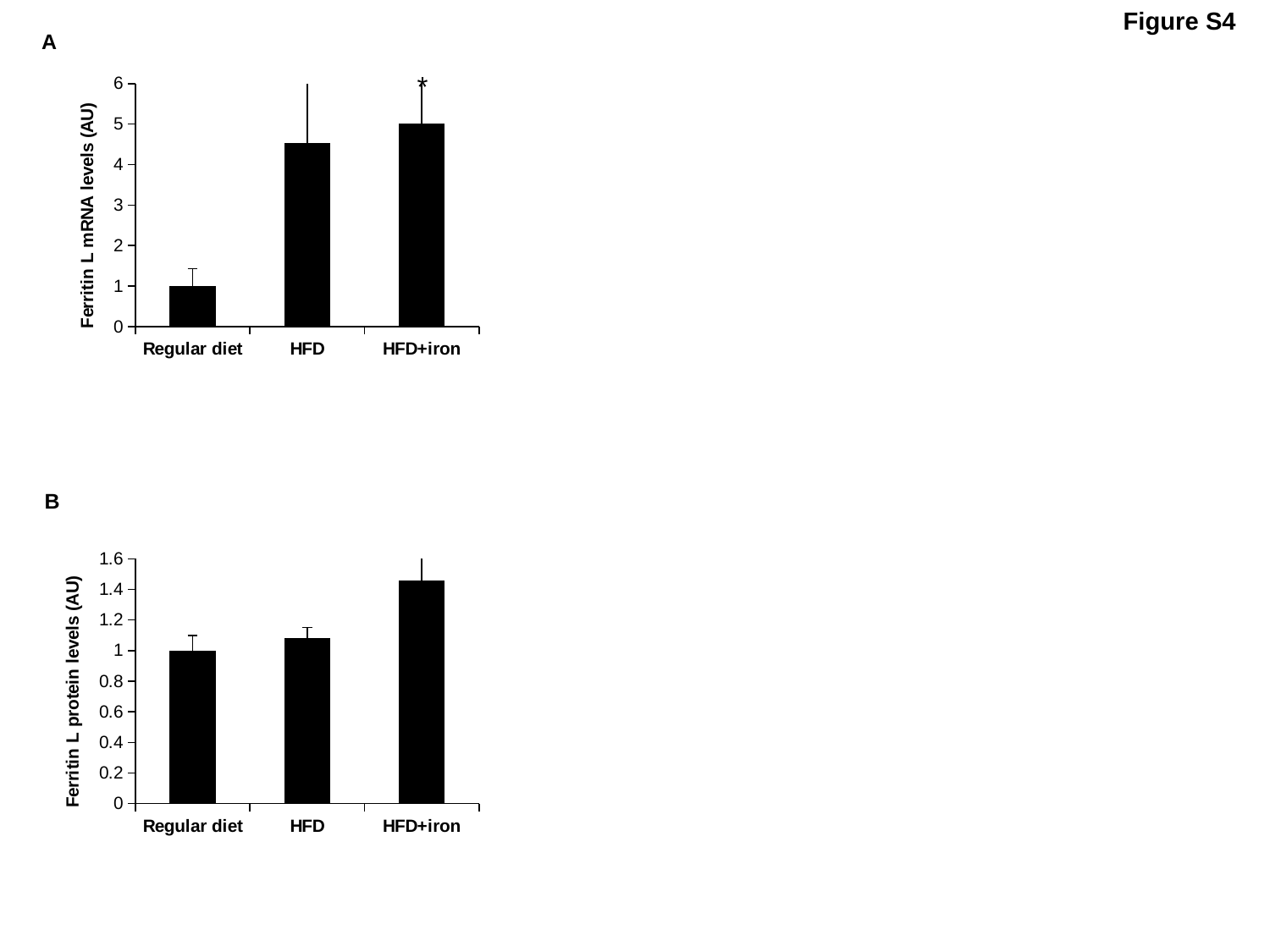

Figure S4
### Chart
| Category | |
|---|---|
| Regular diet | 1.0 |
| HFD | 4.52866242038217 |
| HFD+iron | 5.006369426751592 |A
B
### Chart
| Category | media |
|---|---|
| Regular diet | 1.0 |
| HFD | 1.08 |
| HFD+iron | 1.46 |
