# Supplementary material for: High Fat Diet Subverts Hepatocellular Iron Uptake Determining Dysmetabolic Iron Overload
Source: PLoS One. 2015 Feb 3;10(2):e0116855. doi: 10.1371/journal.pone.0116855 (PMC4315491; doi:10.1371/journal.pone.0116855)
Supplement: S6 Fig — HepG2 hepatocytes were incubated with LnA (0.025 mM), SA (0.025 mM), EA (0.1 mM), LA (0.2 mM) or with FAC (150 μM) alone or in combination with FFA for 24 hours. A) Intracellular iron concentration was measured by atomic absorption spectrometry. B) TfR-1 mRNA levels evaluated by qRT-PCR. C) TfR-1 protein levels were evaluated by Western Blotting. β-actin is shown as the loading control. D) Densitometric analysis of TfR-1 protein levels. Results are mean values of three independent experiments, each experimental condition was evaluated in triplicate. Values are expressed as means±SD. AU, arbitrary units. *p<0.05 vs. controls (normalized for β-actin). (PPTX) [file pone.0116855.s006.pptx]

## Slide 1
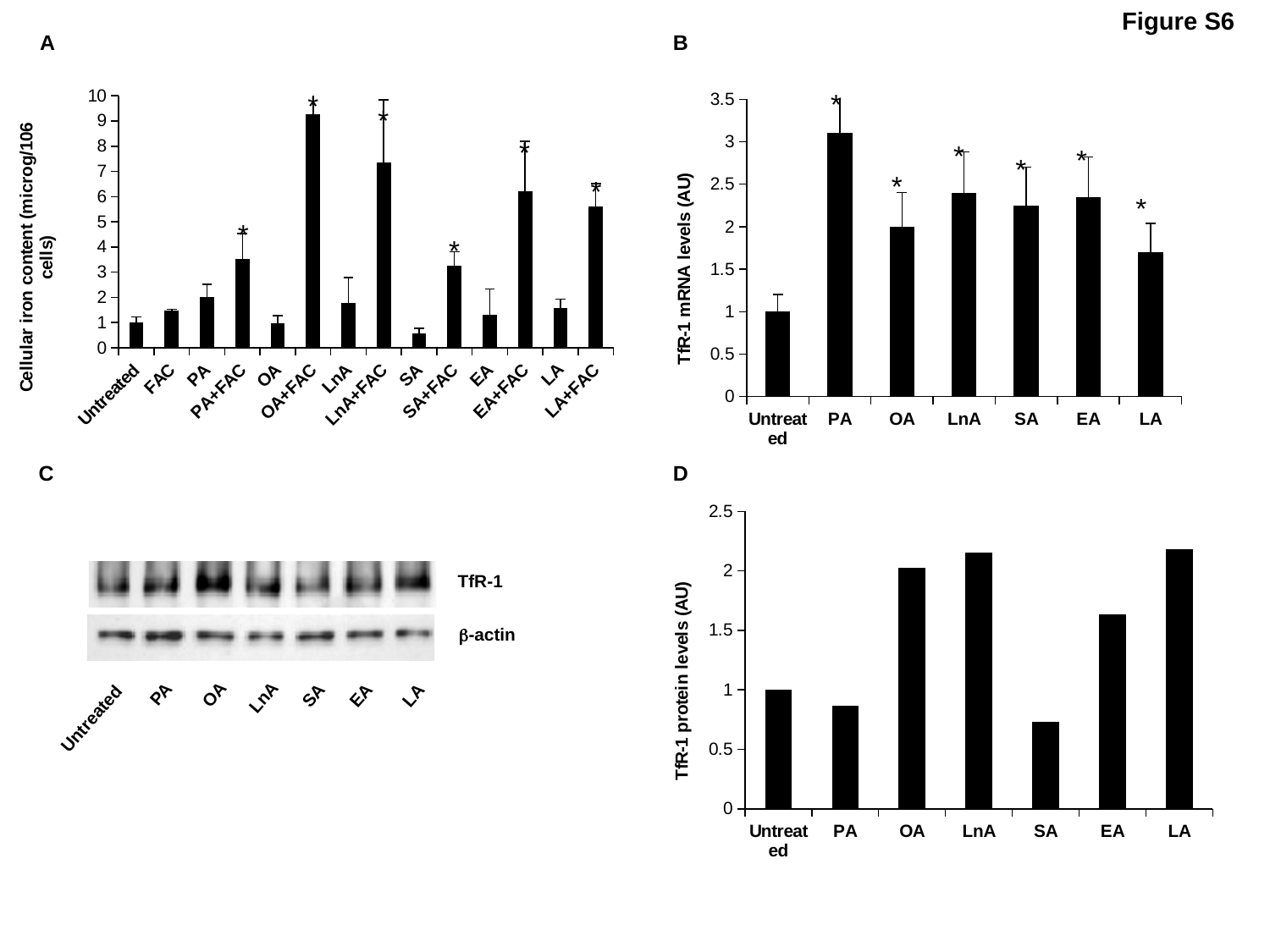

Figure S6
A
B
### Chart
| Category | Media |
|---|---|
| Untreated | 1.0 |
| FAC | 1.48 |
| PA | 2.02 |
| PA+FAC | 3.53 |
| OA | 0.98 |
| OA+FAC | 9.26 |
| LnA | 1.79 |
| LnA+FAC | 7.34 |
| SA | 0.58 |
| SA+FAC | 3.27 |
| EA | 1.3 |
| EA+FAC | 6.2 |
| LA | 1.59 |
| LA+FAC | 5.6 |*
*
*
*
*
*
*
*
*
*
*
*
### Chart
| Category | Media |
|---|---|
| Untreated | 1.0 |
| PA | 3.1 |
| OA | 2.0 |
| LnA | 2.4 |
| SA | 2.25 |
| EA | 2.35 |
| LA | 1.7 |C
D
### Chart
| Category | ratio/actin |
|---|---|
| Untreated | 1.0 |
| PA | 0.86791446201813 |
| OA | 2.024920686892822 |
| LnA | 2.15706100438153 |
| SA | 0.728439581457313 |
| EA | 1.630738119312437 |
| LA | 2.178819184947466 |
TfR-1
b-actin
PA
SA
EA
LA
OA
LnA
Untreated
